# Recruiting refugees to reduce labour shortages in health care professions: experimental evidence on the potential of foreign-language outreach on social media

**DOI:** 10.1186/s12960-024-00933-w

**Published:** 2024-07-03

**Authors:** Jasper Tjaden, Miriam Seuthe, Sebastian Weinert

**Affiliations:** 1 Faculty of Economic and Social Sciences, University of Potsdam, Potsdam, Germany; 2 Fuerst Donnersmark-Foundation Berlin, Berlin, Germany

**Keywords:** Social media, Facebook, Nursing, Refugee, Migrant, Recruitment

## Abstract

**Background:**

Many high-income countries are grappling with severe labour shortages in the healthcare sector. Refugees and recent migrants present a potential pool for staff recruitment due to their higher unemployment rates, younger age, and lower average educational attainment compared to the host society's labour force. Despite this, refugees and recent migrants, often possessing limited language skills in the destination country, are frequently excluded from traditional recruitment campaigns conducted solely in the host country’s language. Even those with intermediate language skills may feel excluded, as destination-country language advertisements are perceived as targeting only native speakers. This study experimentally assesses the effectiveness of a recruitment campaign for nursing positions in a German care facility, specifically targeting Arabic and Ukrainian speakers through Facebook advertisements.

**Methods:**

We employ an experimental design (AB test) approximating a randomized controlled trial, utilizing Facebook as the delivery platform. We compare job advertisements for nursing positions in the native languages of Arabic and Ukrainian speakers (treatment) with the same advertisements displayed in German (control) for the same target group in the context of a real recruitment campaign for nursing jobs in Berlin, Germany. Our evaluation includes comparing link click rates, visits to the recruitment website, initiated applications, and completed applications, along with the unit cost of these indicators. We assess statistical significance in group differences using the Chi-squared test.

**Results:**

We find that recruitment efforts in the origin language were 5.6 times (Arabic speakers) and 1.9 times (Ukrainian speakers) more effective in initiating nursing job applications compared to the standard model of German-only advertisements among recent migrants and refugees. Overall, targeting refugees and recent migrants was 2.4 (Ukrainians) and 10.8 (Arabic) times cheaper than targeting the reference group of German speakers indicating higher interest among these groups.

**Conclusions:**

The results underscore the substantial benefits for employers in utilizing targeted recruitment via social media aimed at foreign-language communities within the country. This strategy, which is low-cost and low effort compared to recruiting abroad or investing in digitalization, has the potential for broad applicability in numerous high-income countries with sizable migrant communities. Increased employment rates among underemployed refugee and migrant communities, in turn, contribute to reducing poverty, social exclusion, public expenditure, and foster greater acceptance of newcomers within the receiving society.

**Supplementary Information:**

The online version contains supplementary material available at 10.1186/s12960-024-00933-w.

## Background

As many other OECD countries, the German economy is suffering from massive labour shortages [8]. The Federal Office for Building and Regional Planning estimates that the potential workforce will decrease by 3 million between 2020 and 2040 [2]. According to employer surveys, the percentage of companies in Germany stating that their business activities are currently hampered by a shortage of skilled workers is 40–50% in 2023 [3].

The healthcare sector in Germany is particularly affected by the shortage of skilled workers. The consulting firm PWC estimates that there will be a shortage of 1.8 million nurses in the healthcare sector in 2035. This would correspond to a relative shortage of 35.4% of all nursing staff currently required in Germany [24]. Combating the nursing shortage is an official goal of the current German government [4].

Three basic strategies are commonly discussed to combat the staff shortage in the healthcare sector, particularly in the nursing professions:

Firstly, attempts are being made to recruit trained specialists abroad [21]. In view of an ageing population and low birth rates, it is estimated that Germany needs a net immigration of up to 400,000 people per year to keep the labour force potential constant in the long term [16]. In view of low immigration figures, it is doubtful that immigration will significantly ease the problem of skilled labour demand.

Secondly, further developments in the field of digitalization and robotics are seen as having the potential to reduce staffing requirements in the care and nursing sector through efficiency gains [22, 23, 25]. This is based on the hope that digital management solutions and robotics can relieve staff in individual work steps or—for example in the area of medication administration—even replace them. This development is in its early stages and faces resistance from employers, staff and patients.

The third strategy is to attract people who are currently not fully participating in the labour market. This “hidden reserve”, i.e. people who are not employed but not registered as unemployed, as well as people who are registered as unemployed, present a potential audience for recruiters which is less costly than recruitment abroad or digitalization.

Migrants are a group with an above-average level of unemployment compared to the general population in Germany, and this applies to a greater extent to the group of refugees [7]. At the end of 2022, Germany recorded 3 million asylum seekers and refugees living in Germany, including over 1.14 million Ukrainian nationals, 674.000 Syrian nationals, 286.000, Afghan nationals, 211.000 Iraqi nationals [11].

Refugees and asylum seekers offer great potential for the recruitment of healthcare and nursing staff. On the one hand, migrants and refugees are, on average, younger than unemployed Germans and therefore have a higher potential for training and employment [11].

However, refugees face many obstacles when searching for jobs, including bureaucratic access to work permits, slow and incomplete recognition of foreign qualifications, a lack of language skills and a lack of labour market information [5, 15]. Limited language skills make it harder to meet language requirements for the job, but also present obstacle for finding and assessing suitable job postings [28]. In traditional media, poster, print or radio campaigns are only launched in the main language of the country to reach the largest audience possible. This practice excludes foreign-language workers.

In this intervention study, we are testing the potential of a targeted, low-cost, language-specific approach via the social media platform Facebook. In cooperation with the Fuerst Donnersmark Foundation Berlin, a foundation proving care for disabled persons, we disseminate job offers for nursing staff in Berlin and Brandenburg in Arabic, Ukrainian and German. The campaign was a real recruitment effort and no deception of users was involved.

Leaders in the nurse profession highlighted the widespread use and cost effectiveness of online media advertising for attracting staff already 15 years ago [6], however, systematic academic evidence of online media recruitment has been lacking [30] . The literature on social media, nursing and human resources is predominantly focused on recruiting nurses as study participants via social media [19, 29]. Reviews of policies and interventions mention examples of small-scale pilot projects which aim to recruit migrants for health professions, for example in the Netherlands and Germany [17], however, no evidence regarding programme effects is available. Health care providers communicate actively on social media platforms [14], yet recruitment appears to play a minor role. Analysing the Facebook content of 17 major US-based health care institutions, Kordzadeh and Young [18] found that less than 1% of posts were related to staff recruitment. Most commonly health care providers use social media, such as Facebook to “engage patients or consumers, build greater brand recognition, and attract new customers” [26]. Studies show that health care providers may use social media to screen persons who have already applied [1, 10, 14] and that social media presence may affect the brand recognition of health care providers [9].

In this study, we demonstrate that foreign language-sensitive advertisements led to a significantly increased response by Arabic and Ukrainian speakers. This shows that simple language-sensitive outreach can play a role in attenuating staff shortages in the nursing sector, especially in countries with sizable migrant and refugee populations.

## Methods

### Study design

The study was designed as an online experiment using Facebook as the delivery platform [20, 27]. According to the parent company Meta, 40–47 million people in Germany can be reached via the Facebook advertising platform, which corresponds to around 56.5% of the German population. Meta estimates that there are around 5 million users in Germany who do not use German as their main language on Facebook. This corresponds to 10.6% of all Facebook users in Germany and is comparable to the proportion of first-generation migrants in the German population [11].

**Fig. 1:**
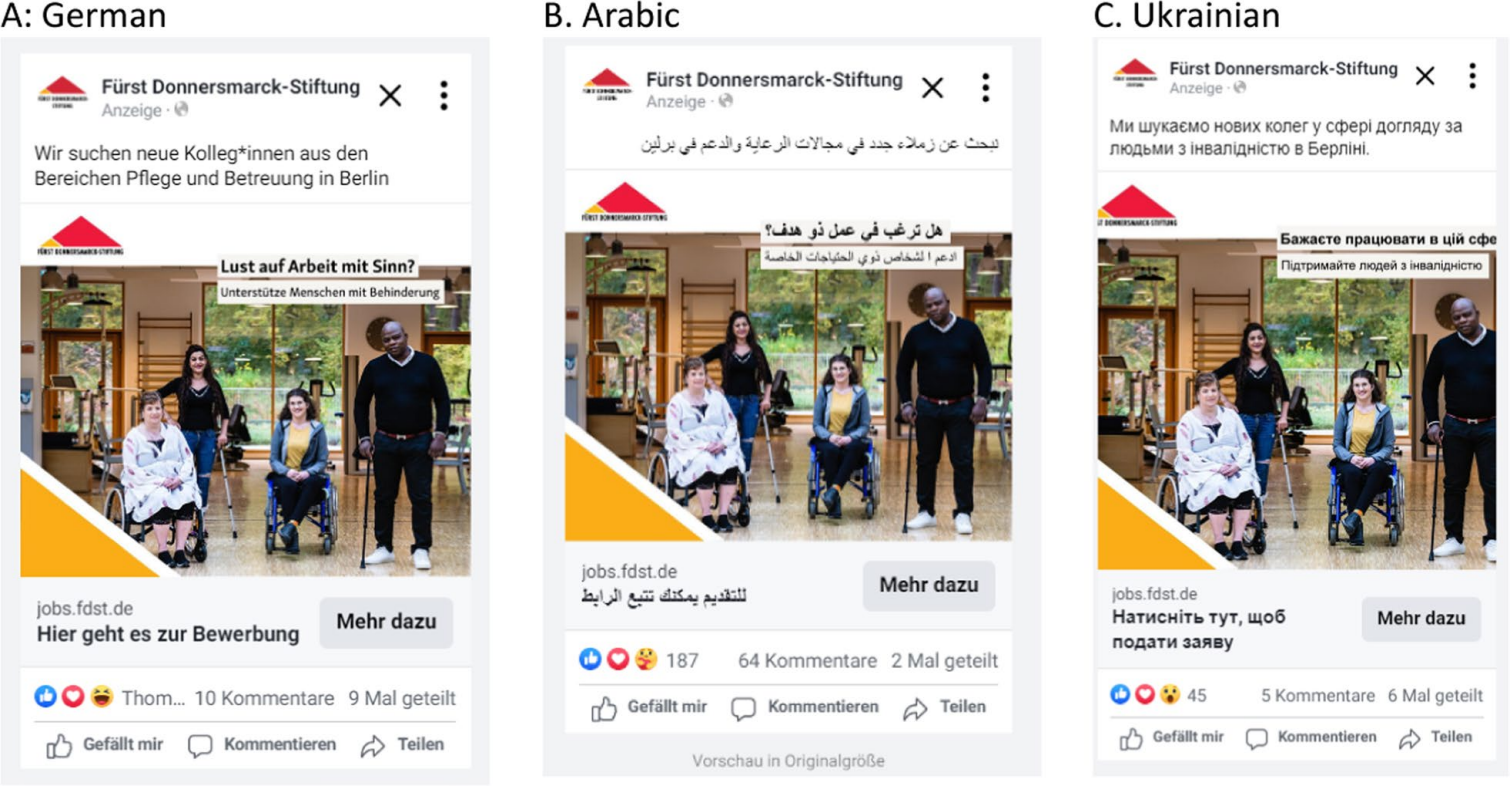
Ad sets

Via the platform’s AB test tool, we launch and compare various advertisements varying by the language of the advertisement and the language used by target audiences. The AB test allows campaign operators to experimentally test the effectiveness of advertisements against each other. In AB tests, different variants of an ad are placed for the same target group. In our case, the same ad was displayed once in German and once in the language of origin (Arabic and Ukrainian) (see Fig. 1). In addition, a German-speaking target group was used as a comparison group (see Table 1).

**Table 1:** Experimental design

Target group	Experiment 1: Arabic-speaking Facebook users		Experiment 2: Ukrainian-speaking Facebook users		German-speaking Facebook users
Ad group	Ad in Arabic	Ad in German	Ad in Ukrainian	Ad in German	Ad in German

The AB test randomly selects users who fall into the target group and ensures that only one variant of the ad is shown to each user. Both test groups are comparable as required by the AB test assignment into control and treatment groups. Facebook limits the possibility to validate balance across groups to a few variables. Groups in our experiment were balanced in terms of sex and age (see Table S1). At the end of the campaign, a series of metrics (see below) are compared between the two groups to determine which version of the ad was more successful.

The campaign ran in Berlin and Brandenburg from August 30 to September 21, 2023 (a total of 22 days) and reached a total of 116,118 users, approximately 7550 per day. The total expenditure for placing the ads on Facebook amounted to €1412.

The job advertisement was developed in collaboration with the Fuerst Donnersmark Foundation according to the corporate design of the organization (FDST). The campaign was a real recruitment initiative, and no deception of users was involved.

The foundation's recruitment team developed a separate application form for German, Arabic and Ukrainian-speaking applicants in their respective languages.

The campaign was designed to encourage users to visit the FDST website by clicking on the provided link. There, they first receive an overview of the job requirements and further information on the working conditions. Another click on “Start application” begins the online application process (see supplementary materials, Figure S1 and S2). It is important to note that individually created URLs ensure that only users exposed to the FB campaign can access the foundation’s website via the Facebook link.

### Measurements

The Facebook advertising platform provides ad operators with a wide range of key figures. In our study, we focus on reach, conversion rates and cost–benefit indicators.

Reach is defined by the number of Facebook user profiles that were exposed to the respective ad at least once in their feed. Since the Facebook algorithms consider the relevance of the ad for the target group when placing the ads algorithmically, the absolute number of users reached (given that the budget used is constant) contains information about which ad was able to achieve higher relevance for the respective target group.

Secondly, we evaluate conversion rates in various forms. Conversion measures the number of users who have clicked on the advertised link in the placed ad (i.e. “link clicks”). The link leads to the advertisement on the FDST website. The link click rate is measured as the number of link clicks per 1000 people reached. Further key figures are collected for the subsequent steps in the application process: after clicking on the link, users are taken to the foundation's website. They then have the option of clicking on “Start application”. This key figure therefore refers to the number of applications started per 1000 users reached on Facebook.

Another focus is on the cost–benefit analysis. This analysis compares the euros spent (reported by the Facebook advertising platform) per link click, per visit to the application form and per application sent. The unit costs result from dividing the overall expenditure for each advertisement by the number of users with successful outcomes. The cost indicator is particularly meaningful to compare across the three language groups as it considers that more money has to be spent on target groups for whom the advertisement appears less relevant in order to achieve a response in the click rate.

As a final step, we examine potential variations in the key indicators above depending on the gender and age of the users.

The statistical analysis is a descriptive comparison of the rates between the different language groups. Differences in the rates are tested for statistical significance using Pearson’s Chi-squared test and reported in the figure or table caption. For robustness, we estimate the main treatment effect models once with, and once without, adjusting for age and sex accounting for unlikely confounding in our experiments (see Table S2). The results remain consistent.

## Results

### Reach

The campaign reached a total of 116,118 people in the Berlin/Brandenburg area within a 22-day period, including 43,288 Arabic speakers and 19,326 Ukrainian speakers. Facebook estimates that there are approximately 35,000 active Ukrainian speakers and 115,000 active Arabic-speaking Facebook users per month in the Berlin/Brandenburg area. This means that the campaign was able to reach approximately 38% of Arabic-speaking users and 55% of Ukrainian-speaking users in period of 22 days. Users saw one of the job advertisements between 2 and 5 times in their feed.

In total, the campaign led to 148 visitors to the FDST application form and 6 submitted applications (see Table 2).

**Table 2:** Overview of ad performance measured in total reach, clicks, visits and applications

Ad group Speakers (ad language)	Reach (Total)	Link clicks	Visits to application website	Applications
All	116,118	3535	148	6
Arabic	28,397	956	94	5
Arabic (DE)	14,891	497	8	0
German	53,356	1147	18	0
Ukrainian	11,223	560	22	1
Ukrainian (DE)	8103	375	6	0

Data collected by the authors in 2023 via Facebook

### Conversions

**Fig. 2:**
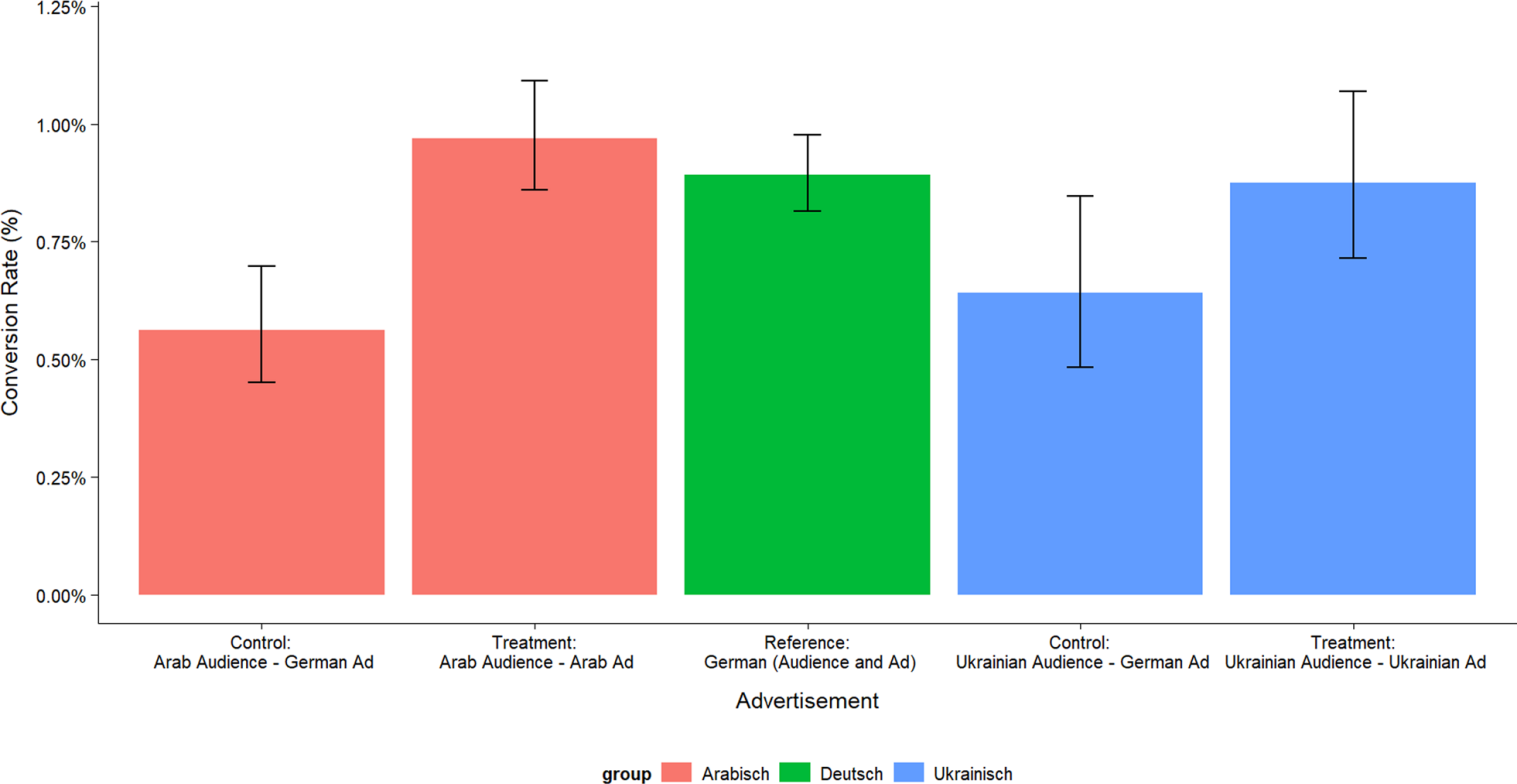
Pearson's Chi-squared test statistic yield statistically significant difference in rates between treatment and control for the Arab-speaking audience (*p*-value < 0.000); difference between treatment and control for the Ukrainian-speaking group is significant at the 10% level (*p*-value = 0.068). Note: Link-clicks per 100 reached users (in %)

The success of the campaign varied for the individual language groups. Figure 2 shows the conversion rates (the so-called “click-through rate”), i.e. the proportion of all users who clicked on the job ad on Facebook (link clicks) among those who saw the ad (reach). The reach is divided by the frequency of the ad display per user to take into account that some people have seen the same ad more often than others.

Figure 2 shows that the click rates for ads in the language of origin are significantly higher than for ads in German, and, on average, similar to the rate for German-speaking users.

For Arabic-speaking users, 9.7 users per 1000 users reached click on the Arabic job ad compared to 5.6 Arabic-speaking users who are shown an ad in German. The ad in the language of origin is 73% more successful than the German-language ad. Even if the advantages of the language of origin were to be expected, the strong effects show the potential of a targeted approach in the language of origin.

For Ukrainian-speaking users, 8.8 users per 1000 users reached click on the ad in the language of origin compared to 6.4 users for the ad in German, an effect of 38%.

Statistical tests show that the differences are statistically significant in both cases (see *p*-values in the note under Fig. 2).

**Fig. 3:**
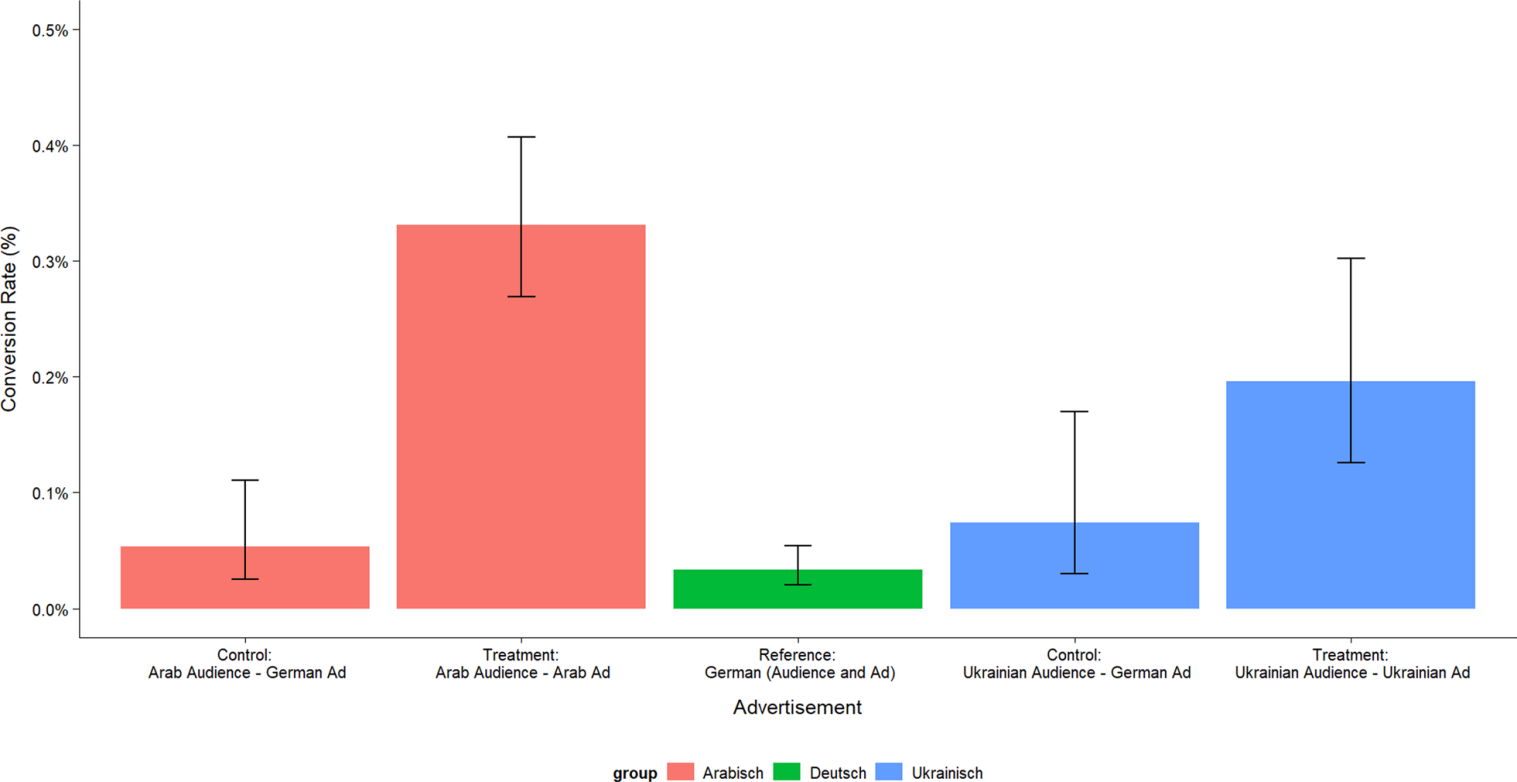
Pearson’s Chi-squared test statistic yields statistically significant difference in rates between treatment and control for the Arab-speaking audience (*p*-value < 0.000); difference between treatment and control for the Ukrainian-speaking group is significant at the 5% level (*p*-value = 0.034). Difference between treatments and the German reference group both statistically significant at *p* < 0.000. Note: Visits to job application website per 100 reached users

Figure 3 shows the conversion rate as the number of users who click on the application form on the foundation’s website after they have been able to view all the information about the position. Here we see even clearer effects of addressing users in their language of origin.

Out of 1000 Arabic-speaking users reached who are shown an Arabic ad, an average of 3.3 people start an application compared to 0.5 Arabic-speaking users who receive the same ad in German. The Arabic ad is therefore 560% more successful than the German ad.

For every 1000 Ukrainian-speaking users reached, an average of 2.0 people who received a Ukrainian ad and 0.7 people who received a German ad started an application. This corresponds to an effect of 186%.

Also noticeable was a much higher level of interaction (comments, shares, likes, etc.) among Arabic (1.8 times) or Ukrainian-speaking (2.4 times) users compared to the German ads. On the one hand, this indicates the high level of user interest in the ad, but on the other hand it also has a positive effect on the reach of the ad, as posts with high interaction rates are automatically displayed more frequently by Facebook.

We observe extreme differences in comparison to the German-speaking reference group. The job advertisement appears to have a greater relative appeal to Arabic and Ukrainian speakers on Facebook than to German speakers, even when foreign speakers are addressed in German. This shows that immigrants and refugees are interested in jobs in the care sector and that there is an untapped potential workforce in this group. The number of German-speaking people who start an application is 8 times lower compared to Arabic-speaking people and 5 times lower compared to Ukrainian-speaking people.

### Cost–benefit analysis

The strong advantages of the language of origin-specific approach are also reflected in the cost–benefit calculation. Table 3 shows how many euros were spent to achieve one click, one application started and one complete application. Since the German-language ads did not lead to a complete application in any group, the calculations are only possible for the Arabic-speaking and Ukrainian-speaking users.

**Table 3:** Cost per click, job website visit and completed application

Ad group (ad language)	Euro per click	Euro per initiated application	Euro per completed application
Arabic	0.84	2.47	46.4
Arabic (German)	2.82	29.5	
German	0.99	26.22	
Ukrainian	2.4	10.73	236
Ukrainian (German)	4.55	39.33	

For German-speaking users, €26 had to be spent to achieve one application started (not submitted). For Arabic-speaking and Ukrainian-speaking users, who were addressed in their respective languages of origin, the costs amounted to €2.4 and €10.7. Addressing Arabic-speaking and Ukrainian-speaking users is therefore 10.8 and 2.4 times “cheaper” than achieving the same response from German-speaking users.

The Arabic-language ads generated a total of 5 completed applications, €46 per application. The Arabic-language ads achieved a total of 1 completed application, 236€ per application.

It is difficult to compare the costs of Facebook job advertisements to other marketing channels. Anecdotally, FDST reported costs between 400 and 900€ per month on other online job websites, and between 300 and 900€ per week in print media. The reach of these channels is unknown and the cost per application was not tracked. However, the Facebook campaign appeared qualitatively cheaper than alternative approaches, particularly print media.

### Subgroup analysis

Further analyses suggest that the success of the ads does not differ significantly according to the gender of the user. The conversion rates are not statistically significantly different across all ad groups. However, there are differences in the number of users reached. It is noticeable here that more men than women were reached in the Arabic-speaking group. The ratio is the other way around in the Ukrainian and German-speaking groups.

However, stronger differences can be observed regarding the age structure of the users. In line with the age structure of Facebook users overall, the results show that the ads are more successful among older cohorts. This trend can be observed across all language groups. This means that even among non-German-speaking users, Facebook campaigns have less potential to appeal to younger cohorts under the age of 35.

## Discussion

The pilot study shows that online campaigns addressing refugees in their mother tongue is substantially more effective and cheaper than the standard approach in the language of the host country. While this finding appears intuitive, evidence on the size of the effect was still missing. We find that language-adequate adverts are 10.8 and 2.4 times “cheaper” than German ads. We also find that addressing foreign-language communities results in more positive responses to job advertisements compared to the German reference group, possibly reflecting higher unemployment rates among migrant and refugee communities and the fact that nursing jobs in the care sector offer fewer entry obstacles in terms of qualifications. Social media may be particularly well suited for addressing refugee communities which includes many persons without formal qualifications who are not members of dedicated high-skill job search platforms such as LinkedIn.

Given the results, it is surprising that employers are currently not engaging with foreign-language communities much. Faced with high and growing labour shortages, employers will need to be creative and experiment with new recruiting channels. This study provides a first piece of evidence of the potential of reaching and engaging migrants and refugees through origin-language advertising through social media. Increasing employment rates among migrant and refugees would have several positive spillover effects such as higher household income and wellbeing, acquisition of language and social contacts through work, as well as acceptance of newcomers among the receiving society’s population.

Targeted recruitment approaches can only be one element to a broader solution. To unfold their full potential, they need to be embedded in supporting structures of health care providers. The evidence base on such supporting structures is still limited, yet plausible measures include: (1) offering on-the-job training for newcomers as migrants and refugees often do not have the country-specific certified degrees or trainings, and (2) relaxing formal hiring requirements especially related to language skills and, instead, offering supplementary vocational language training.

Our study is not without limitations. Firstly, the Facebook platform cannot directly identify refugees as a legal residence category. Narrowing down the target group by language (Arabic, Ukrainian) is therefore merely an appropriate approximation. It can be assumed that people who are in Germany and use Facebook in a foreign language have been in Germany for a few years, as they have not yet acquired an advanced knowledge of German in order to change their user language. The majority of Arabic and Ukrainian speakers who have immigrated to Germany in the last 10 years are refugees [11].

Secondly, although the study design is similar to a randomized field experiment, there are differences related to the placement algorithm on Facebook which are out of the control of researchers [12, 13]. This complicates a causal interpretation of the effects, as endogeneity cannot be ruled out completely. We have presented numerous key outcomes that mitigate endogeneity problems as much as possible and further demonstrated balance between groups in terms of sex and age. Furthermore, the Facebook AB testing tool had the advantage of being easily applicable in practice by non-scientific personnel. This facilitates replication for researchers and scalability and uptake among practitioners.

Third, our study was only able to assess one campaign via one specific social media platform (Facebook). As user demographics and popularity of different platforms change, future research should explore the use of alternative platforms such as TikTok, Instagram or dedicated job sites such as LinkedIn.

Fourth, while the study showed effective reach and engagement of the target group and the potential of foreign-language outreach, it was unable to disentangle the reasons why many refugees seek more information about the job but refrain from completing an application. Potential explanations such as insufficient compliance with formal requirements (language level, drivers license etc.) or dissuasion by characteristics of the job itself (salary, hours, location) are plausible and deserve further attention. Many more social media users commented on the job ad signalling their interest in the job, than the number of candidates who followed through with an application. Further efforts from the employer could increase applications by following up with interested candidates online and finding flexible solutions.

### Supplementary Information

Additional file 1: Table S1. Number of FB users reached by age and sex. Table S2. Main treatment effects by specification and outcome variable. Figure S1. Screenshot of recruitment website featuring information about advertised job (in Ukrainian). Figure S2. Application form (in German).

## Data Availability

Replication materials are accessible at the Open Science Foundation at xxxx.
