# Supplementary material for: Recruiting refugees to reduce labour shortages in health care professions: experimental evidence on the potential of foreign-language outreach on social media
Source: Hum Resour Health. 2024 Jul 3;22:48. doi: 10.1186/s12960-024-00933-w (PMC11223288; doi:10.1186/s12960-024-00933-w)
Supplement: Supplementary file 1 — Additional file 1: Table S1. Number of FB users reached by age and sex. Table S2. Main treatment effects by specification and outcome variable. Figure S1. Screenshot of recruitment website featuring information about advertised job (in Ukrainian). Figure S2. Application form (in German). [file 12960_2024_933_MOESM1_ESM.docx]

**Supplementary Materials**

**Table S1: Number of FB users reached by age and sex**

|  | **Ukrainian sample** | | | | **Arabic Sample** | | | | **German Sample** | |
| --- | --- | --- | --- | --- | --- | --- | --- | --- | --- | --- |
|  | *N* | *N* | *Row %* | *Row %* | *N* | *N* | *Row %* | *Row %* | *N* | *Row %* |
|  | German | Ukrainian | German | Ukrainian | German | Arabic | German | Arabic | German | German |
| **Age** | | | | | | | | | | |
| 65+ | 896 | 1252 | 0.11 | 0.11 | 1212 | 1384 | 0.08 | 0.05 | 13007 | 0.24 |
| 55-64 | 1442 | 2196 | 0.18 | 0.20 | 1952 | 2760 | 0.13 | 0.10 | 15343 | 0.29 |
| 45-54 | 1986 | 3100 | 0.25 | 0.28 | 2320 | 5367 | 0.16 | 0.19 | 8911 | 0.17 |
| 35-44 | 2172 | 3208 | 0.27 | 0.29 | 3128 | 7591 | 0.21 | 0.27 | 5136 | 0.10 |
| 25-34 | 1260 | 1224 | 0.16 | 0.11 | 4280 | 9407 | 0.29 | 0.33 | 5584 | 0.10 |
| 18-24 | 348 | 244 | 0.04 | 0.02 | 2000 | 1888 | 0.13 | 0.07 | 5376 | 0.10 |
| **Sex** | | | | | | | | | | |
| female | 5818 | 8684 | 0.72 | 0.77 | 5096 | 9704 | 0.34 | 0.34 | 37614 | 0.71 |
| male | 2264 | 2516 | 0.28 | 0.22 | 9736 | 18614 | 0.65 | 0.66 | 15344 | 0.29 |
| unknown | 22 | 24 | 0.00 | 0.00 | 60 | 80 | 0.00 | 0.00 | 323 | 0.01 |

**Table S2: Main treatment effects by specification and outcome variable**

|  | **Ukrainian** | | | | **Arabic** | | | |
| --- | --- | --- | --- | --- | --- | --- | --- | --- |
| **Outcome** | **b** | **p-value** | **b** | **p-value** | **b** | **p-value** | **b** | **p-value** |
| Link  clicks | -0.314 | 0.006 | -0.307 | 0.008 | -0.550 | 0.000 | -0.553 | 0.000 |
| Website visits | -0.974 | 0.034 | -1.258 | 0.000 | -1.821 | 0.000 | -2.112 | 0.000 |
| Controls  (sex, age) |  |  | yes | yes |  |  | yes | yes |

**Figure S1. Screenshot of recruitment website featuring information about advertised job (in Ukrainian)**

**
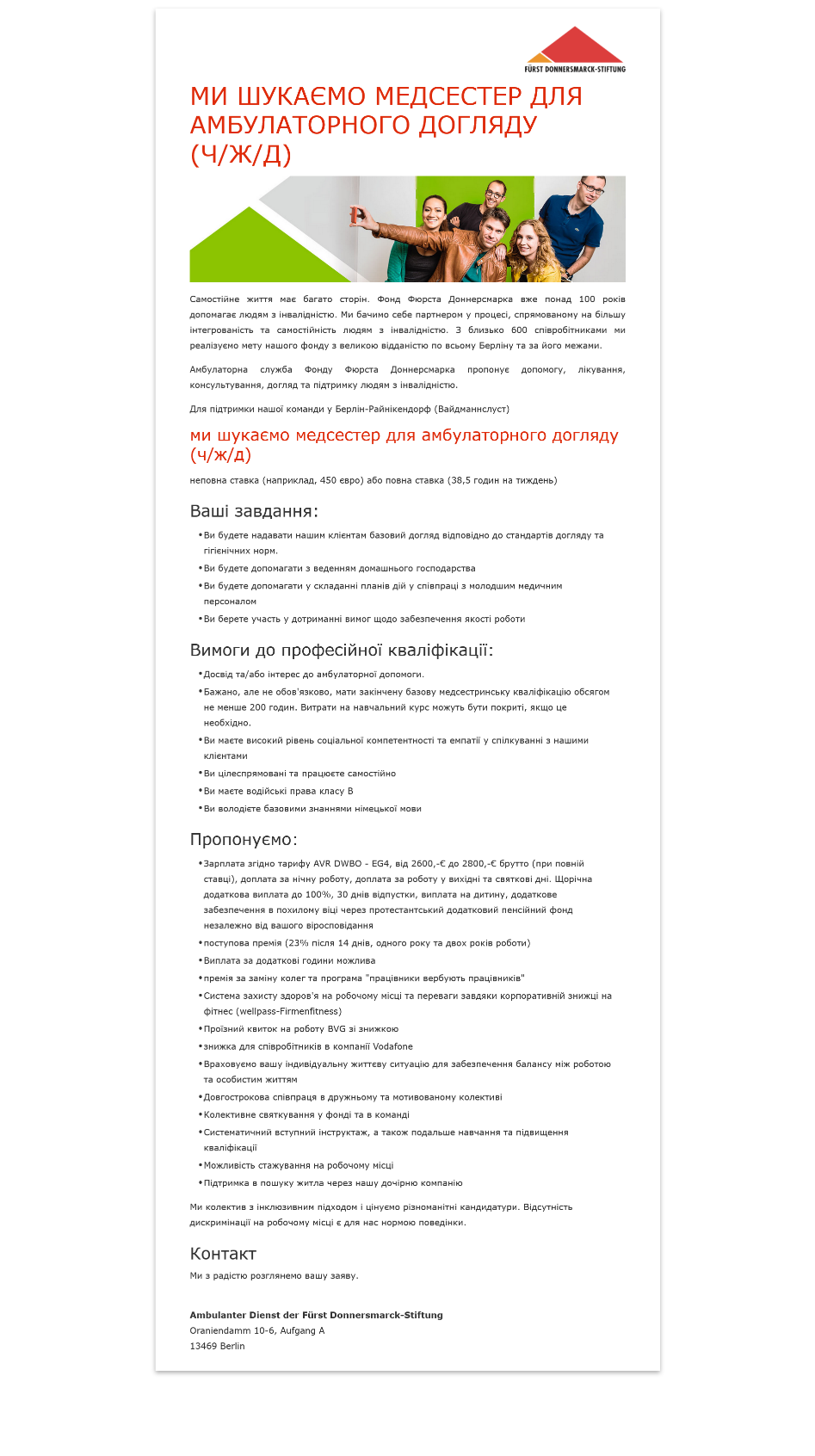
**

**Figure S2. Application form (in German)**

**
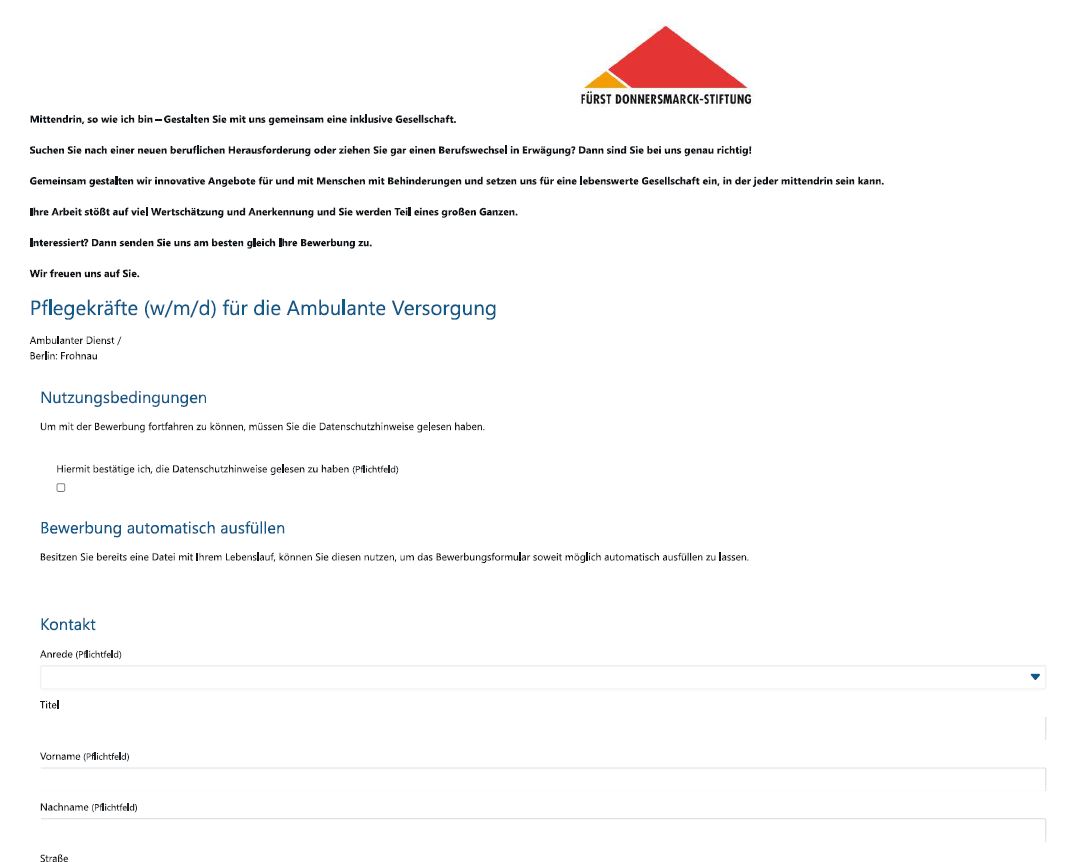
**
